# Prognostic value of whole-body SUVmax of nodal and extra-nodal lesions detected by ^18^F-FDG PET/CT in extra-nodal NK/T-cell lymphoma

**DOI:** 10.18632/oncotarget.13873

**Published:** 2016-12-10

**Authors:** Jin-Hua Liang, Chong-Yang Ding, Robert Peter Gale, Li Wang, Ji Xu, Xiao-Yan Qu, Lei Fan, Tian-Lv Li, Jian-Yong Li, Wei Xu

**Affiliations:** ^1^ Department of Hematology, The First Affiliated Hospital of Nanjing Medical University, Jiangsu Province Hospital, Nanjing, China; ^2^ Department of Nuclear Medicine, The First Affiliated Hospital of Nanjing Medical University, Jiangsu Province Hospital, Nanjing, China; ^3^ Haematology Research Centre, Division of Experimental Medicine, Department of Medicine, Imperial College London, London, United Kingdom; ^4^ Collaborative Innovation Center for Cancer Personalized Medicine, Nanjing Medical University, Nanjing, China

**Keywords:** PET/CT, extranodal NK/T-cell lymphoma, prognosis, SUVmax

## Abstract

We analyzed data from 54 newly-diagnosed persons with extra-nodal natural killer/T-cell (NK/T) lymphoma, who had a pretreatment ^18^F-FDG PET/CT study, to determine whether the sum of SUVmax of all the nodal and extra-nodal lesions predicted progression-free survival (PFS) and/or overall survival (OS). Three models (WB1SUVmax, WB2SUVmax, WB3SUVmax) based on the basis of the sum of SUVmax of the whole-body SUVmax of 11 nodal and 10 extra-nodal lesions were tested. The discrimination value of these models was evaluated using time-dependent receiver-operator characteristic (ROC) curves and corresponding areas under the curve (AUC) in training and validation cohorts. Findings were validated in an independent cohort of 15 subjects. ROC curve analysis showed the optimal cut-off values for WB1SUVmax, WB2SUVmax and WB3SUVmax were 15.8 (sensitivity 92%, specificity 67%, AUC 0.811; *P*<0.001), 12.7 (sensitivity 96%; specificity 57%; AUC 0.785; *P*<0.001) and 15.8 (sensitivity 88%; specificity 70%; AUC 0.793; *P*<0.001). Multivariate analyses indicated WB3SUVmax was independently associated with PFS (hazard ratio [HR]=3.67, 95% confidence interval [95% CI]=1.19, 11.29; *P*=0.023) and OS (HR= 4.51 [1.02, 19.91]; *P*=0.047). WB3SUVmax calculated based of the sum of the SUVmax of 3 nodal and 10 extra-nodal lesions was significantly associated with PFS and OS.

## INTRODUCTION

Extra-nodal natural-killer (NK)/T-cell lymphoma is a subtype of the mature T- and NK-cell neoplasm in the World Health Organization (WHO) classification [1]. It is common in Asia compared with Western countries: 3-9% of lymphoma *versus* 1% in Western countries [2–4]. NK/T-cell lymphoma is an aggressive lymphoma with 5-year survival <50 percent [5–8].

Several prognostic models have been studied for NK/T-cell lymphoma including the International Prognostic Index [IPI], the Korean Prognostic Index [KPI], the Prognostic Index for T-cell lymphoma, a new model combining the KPI, total protein and fasting blood glucose [8, 9] and a model using data from PET/CT parameters including the maximum standardized uptake value [SUVmax], whole-body metabolic tumour volume [WBMTV] and whole body total lesion glycolysis [WBTLG]) [10–12]. Recently, Kim *et al*. reported combining post-treatment Deauville score on PET/CT with data on Epstein-Barr virus (EBV) DNA predicts treatment-failure in subjects with nasal NK/T-cell lymphoma [13]. Most of these models were tested in persons with early and/or nasal NK/T-cell lymphoma.

Predictive value of SUVmax [10–12] is controversial probably because it measures maximum metabolic rate of some but not all lymphoma sites. In a small study, Kim *et al.* [10] suggested WBMTV and WBTLG might be better survival predictors than SUVmax but these data need confirmation. The SUVmax considers only one voxel. We hypothesized the sum of the SUVmax of all the nodal and extra-nodal tumours measured by ^18^F-FDG PET/CT would better represent lymphoma activity in a person with NK/T-cell lymphoma. To test this hypothesis, we designed three new models on the basis of the whole-body SUVmax of 11 nodal lesions (Waldeyer ring, neck, infra-clavicular, axillary and pectoral, mediastinal, hilar, spleen, para-aortic, mesenteric, lilac, inguinal and femoral) and 10 extra-nodal lesions (upper aero-digestive tract, skin/subcutaneous tissues, central nervous system [CNS] and spinal canal, lung, myocardium, bone and bone marrow, bowel, renal and adrenal, liver and testis). We tested these models in receiver-operator characteristic (ROC) curve analyses to determine which best predicted progression-free survival (PFS) and overall survival (OS) in training (N= 54) and validation (N=15) cohorts.

## RESULTS

### Clinical variables

Clinical variables are outlined in Table 1. Thirty-seven subjects were male. Median age was 46 years (range, 14-85 years). Twenty-seven were Ann Arbor stage-I/II and 20 of whom had extra-nasal cavity type lymphoma. Twenty-six subjects had an elevated serum lactate dehydrogenase (LDH) level and 23 had B-symptoms. Nine subjects had an ECOG score ≥2 and 31 had an IPI score ≥2.

**Table 1:** Subject variables (N=54)

Clinical characteristics	N (%)
**Male**	37 (69%)
**Age ≤60 years**	42 (78%)
**LDH ≤ULN**	26 (48%)
**Ann Arbor stage I/II**	27 (50%)
**Presence of B symptom**	23 (43%)
**ECOG 2-4**	9 (17%)
**Extra-nodal sites ≤1**	32 (59%)
**IPI score 0-1**	23 (43%)

### Values of SUVmax, WB1SUVmax, WB2SUVmax and WB3SUVmax in different groups

Upper aero-digestive tract involvement was present in 41 subjects with median SUVmax of 12.9 (range, 4.5-25.9). Bone and bone marrow involvement were present in 14 subjects with the median SUVmax of 8.0 (range, 2.7-13.5). SUVmax of the other 7 extra-nodal sites were all <20 percent (Table 2).

**Table 2:** Distribution of extra-nodal site involvement

	N	SUVmax (range)
Upper aero-digestive tract	41 (76%)	12.9 (4.5-25.9)
Skin/subcutaneous tissues	7 (13%)	8.5 (2.7-17.1)
Central nervous system and spinal canal	2	8.6 (7.3-10.2)
Lung	8 (15%)	5.3 (1.6-9.3)
Myocardium	2	10.3 (2.4-18.1)
Bone and bone marrow	14 (26%)	8.0 (2.7-13.5)
Bowel	7 (13%)	9.7 (4.0-25.1)
Renal and adrenal	5	11.1 (7.1-15.8)
Liver	4	6.4 (2.1-9.0)
Testis	5 (9%)	8.3 (5.3-15.9)

Median value of WB1SUVmax, WB2SUVmax and WB3SUVmax at diagnosis in the disease progression cohort (N=29) were higher than those in the progression-free group (N=25; *P*<0.0001, *P*=0.002 and *P*<0.001). There were no significant difference in SUVmax (*P*=0.141). Similarly, median value of WB1SUVmax, WB2SUVmax and WB3SUVmax at diagnosis in living subjects (N=30) were significantly higher than values in dead subjects (N=24; *P*<0.001, *P*=0.001 and *P*<0.001). The difference in SUVmax was borderline (*P*=0.072; Table 3).

**Table 3:** Median values of SUVmax, WB1SUVmax, WB2SUVmax and WB3SUVmax in different sub-groups

	PD	≥SD	*P*	Alive	Dead	*P*
SUVmax	14.0 (3.9-25.9)	11.8 (4.5-23.6)	0.141	14.6 (3.9-25.9)	11.9 (4.5-23.6)	0.072
WB1SUVmax	38.8 (3.9-109.0)	17.3 (4.5-54.7)	<0.001	42.0 (3.9-109.0)	19.7 (4.5-65.6)	<0.001
WB2SUVmax	33.6 (3.9-99.2)	17.4 (4.5-58.0)	0.002	36.1 (3.9-99.2)	19.2 (4.5-65.6)	0.001
WB3SUVmax	34.5 (3.9-109.0)	17.1 (4.5-54.7)	<0.001	36.7 (3.9-109.0)	19.4 (4.5-65.6)	<0.001

### Comparison of the models

**Figure 1:**
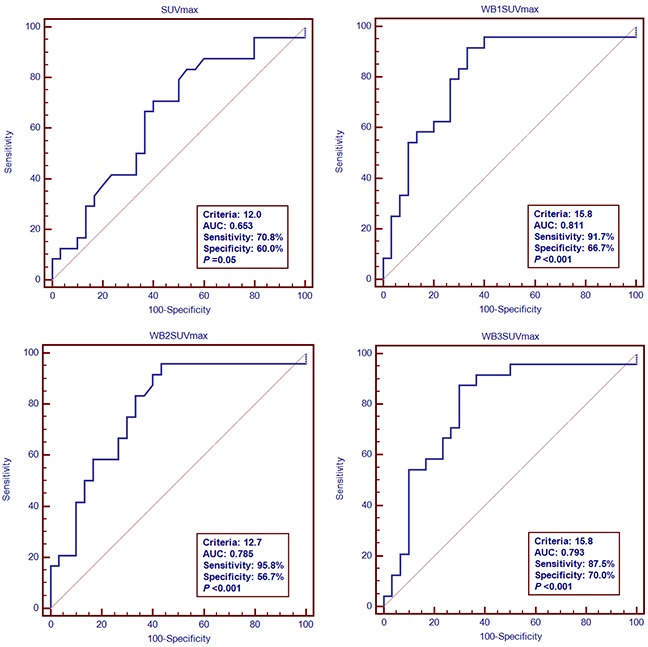
ROC curve analyses of OS

**Figure 2:**
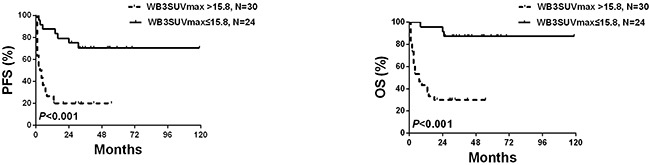
PFS and OS according to WB3SUVmax

We evaluated the predictive accuracy of these models in time-dependent ROC curves which showed optimal cut-off values for SUVmax, WB1SUVmax, WB2SUVmax and WB3SUVmax of 12.0 (sensitivity 71%; specificity 60%; AUC (areas under the curve) 0.653; *P*=0.05), 15.8 (sensitivity 92%; specificity 67%; AUC 0.811; *P*<0.001), 12.7 (sensitivity 96%; specificity 57%; AUC 0.785; *P*<0.001) and 15.8 (sensitivity 88%; specificity 70%; AUC 0.793; *P*<0.001; Figure 1). If we consider OS for the entire follow-up period, the AUCs of SUVmax, WB1SUVmax, WB2SUVmax and WB3SUVmax were 0.653 [0.511, 777], 0.811 [0.681, 0.905], 0.785 [0.653, 0.885] and 0.793 [0.661, 0.891]. Pair-wise comparisons of ROC curves in the models are shown in Supplementary Table S1. Significant improvement in discrimination for WB1SUVmax, WB2SUVmax and WB3SUVmax compared with SUVmax was observed. There were no significant differences between WB1SUVmax, WB2SUVmax and WB3SUVmax and we selected WB3SUVmax which is the simplest of the 3 models to compute for further analyses. Kaplan-Meier PFS and OS cures for the WB3SUVmax model using the optimal cut-off value are shown in Figure 2.

Parameters (sex, age, Ann Arbor stage, LDH level, B-symptom, extra-nodal involvement sites, ECOG score, IPI score and WB3SUVmax) significantly associated with PFS and OS were entered into multivariate analyses. WB3SUVmax >15.8 was significantly associated with PFS (HR 3.67 [1.19, 11.29]; P=0.023) and OS (HR 4.51 [1.02-19.91]; P=0.047; Table 4).

**Table 4:** Uni- and multivariate Cox regression analyses for PFS and OS

	Univariate (PFS)		Multivariate (PFS)		Univariate (OS)		Multivariate (OS)	
Characteristic	HR (95%CI)	P	HR (95%CI)	P	HR (95%CI)	P	HR (95%CI)	P
WB3SUVmax>15.8	5.23 (2.20-12.40)	<0.001	3.67 (1.19-11.29)	0.023	9.66 (2.85-32.76)	<0.001	4.51 (1.02-19.91)	0.047
IPI≥2	7.37 (2.78-19.51)	<0.001	--	--	13.81 (3.22-59.21)	<0.001	--	--
Age>60 years	0.786 (0.32-1.92)	0.596	--	--	0.79 (0.30-2.13)	0.650	--	--
ECOG PS>1	2.07 (0.92-4.65)	0.078	--	--	2.23 (0.88-5.64)	0.091	--	--
LDH>ULN	5.50 (2.33-12.98)	<0.001	--	--	7.24 (2.46-21.38)	<0.001	--	--
Stage III or IV	4.17 (1.89-9.21)	<0.001	--	--	5.62 (2.08-15.17)	0.001	--	--
Extra-nodal sites>1	3.05 (1.48-6.32)	0.003	--	--	5.63 (2.31-13.74)	<0.001	--	--
Gender (male)	0.69 (0.33-1.42)	0.314	--	--	0.71 (0.31-1.63)	0.422	--	--
B symptoms	2.92 (1.30-6.56)	0.010	--	--	3.74 (1.39-10.05)	0.009	--	--

### Validation cohort for WB3SUVmax

**Figure 3:**
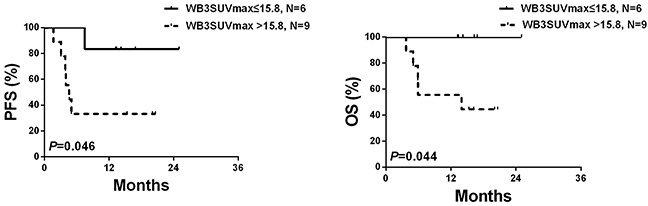
PFS and OS of the validation cohort (N=15) according to WB3SUVmax

Data from the validation cohort confirmed the predictive value of WB3SUVmax for PFS and OS (Figure 3).

## DISCUSSION

The predictive values of PET/CT parameters such as SUVmax, WBMTV and WBTLG in extra-nodal NK/T-cell lymphomas are controversial [10–12, 16–22]. One problem with using SUVmax is that the value represents only the highest metabolic rate in one lymphoma site which may not be representative of metabolic rate in several or all lymphoma sites. As such WBMTV and WBTLG may better represent the sum of lymphoma sites and metabolic activity [10, 20]. Nevertheless, the prognostic value of WBMTV and WBTLG in aggressive lymphomas is controversial [10, 20–22].

Limitations of predictive models for extra-nodal NK/T-cell lymphoma, especially the non-nasal type [2, 10–12, 15–22] led us to study three new prognostic models, WB1SUVmax, WB2SUVmax, WB3SUVmax. The models differ in how they sum SUVmax lymphoma sites but had mostly concordant values. In 40 subjects, WB1SUVmax, WB2SUVmax, WB3SUVmax were similar and in 10 others values were >15.8. We selected WB3SUVmax, because it was the simplest to compute. Although the WB3SUVmax does not represent the total volume and metabolic activity of lymphoma sites, it indicates the sum of the highest metabolic rates of all nodal and extra-nodal lesions. In conclusion, we show WB3SUVmax is a strong predictor of PFS and OS in persons with extra-nodal NK/T-cell lymphoma. Because our validation cohort was small, our conclusion should be tested in other datasets.

## SUBJECTS AND METHODS

### Subjects

The study was approved by the Ethics Committee of the First Affiliated Hospital of Nanjing Medical University and done according to guidelines of Nanjing Medical University. Subjects provided informed consent in accordance with requirements of the Declaration of Helsinki. Between June, 2006 and May, 2014, 54 consecutive subjects with newly diagnosed NK/T-cell lymphoma had a ^18^F-FDG PET/CT scan for staging at our centre. Diagnosis was based on the World Health Organization lymphoma classification [23]. Baseline clinical variables included age, sex, Ann Arbor stage (I-IV), LDH, B-symptoms, extra-nodal disease sites and ECOG performance score. Therapy was with L/P-EMD (L/Peg-asparaginase, etoposide, methotrexate and dexamethasone; Supplementary Table S2). Subjects received a median cycle of 4 (range, 3-6). Thirty subjects also received radiation therapy to residual disease sites. Median follow-up is 45 months (range, 20-120 months).

### ^18^F-FDG PET/CT image acquisition

PET/CT studies were obtained on the following PET/CT devices: Gemini TF64 (Philips), Gemini GXL (Philips), Gemini TF16 (Philips), Discovery LS (GE Healthcare), and Biograph TP16 (Siemens). Subjects with fasting serum glucose <7.0 mmol/L >6 h received IV ^18^F-fluorodeoxyglucose (^18^F-FDG) 3.70−5.55 MBq/Kg. After 60 min whole-body PET/CT imaging was performed with a whole-body CT scan (120 KV and 140 mA) and a whole-body PET (in 3-dimensional mode, 120s/bed position). Acquisition of CT, PET and PET/CT fusion images including cross-section, sagittal-section and coronal-section used CT-based attenuation correction in reconstruction image by an iterative method.

### Image analysis models

The body was divided into 11 nodal regions (Waldeyer ring, neck, infra-clavicular, axillary and pectoral, mediastinal, hilar, spleen, paraaortic, mesenteric, lilac, inguinal and femoral) and 10 extra-nodal regions (upper aero-digestive tract, skin/subcutaneous tissues, central nervous system [CNS] and spinal canal, lung, myocardium, bone and bone marrow, bowel, renal and adrenal, liver and testis) for analyses [2, 15] (Supplementary Table S3). SUVmaxs were evaluated for all subjects. Four models (SUVmax, WB1SUVmax, WB2SUVmax and WB3SUVmax) were used to calculate the whole body SUVmax of the nodal and extra-nodal regions (Table 5).

**Table 5:** Three different models to calculate the whole body SUVmaxs for nodal and extra-nodal regions to predict PFS and survival

Models	Nodal and extra-nodal regions for calculation
**SUVmax**	Maximum standard uptake value of primary lesion
**WB_1_SUVmax**	Whole body SUVmax of 11 nodal and 10 extra-nodal regions
**WB_2_SUVmax**	Whole body SUVmax of 4 nodal^*^ (neck, axillary, inguinal and spleen) and 10 extra-nodal regions
**WB_3_SUVamx**	Whole body SUVmax of 3 nodal regions (superior diaphragm, inferior diaphragm and spleen) and 10 extra-nodal regions

* Four different nodal regions refer to chronic lymphocytic leukemia (CLL)

### Validation cohort

15 subjects receiving PEMD for a median of 4 cycles (range, 2-6) constituted the validation cohort. Median follow-up from diagnosis was 16 mo (range 13- 25 mo).

### Statistical analysis

We used the Epidata 3.10 to establish datasets and verify validity of data-entry twice. The discriminative ability of the model was determined using time-dependent ROC curves and the corresponding AUCs were calculated to assess the predictive accuracy of the models [24]. Differences in AUCs were tested as described [25]. Survival curves were constructed by the Kaplan-Meier method. Log-rank test was used to compare survival times of different groups categorized by the selected best predictive model. Prognostic significances of PET parameter (the best one) and clinical variables (sex, age, IPI score, Ann Arbor stage, LDH level, B-symptoms, ECOG performance and extra-nodal sites) were assessed by univariate analyses. Variables with significant associations were included in multivariate Cox proportional hazards regression analyses. All the statistical analyses used STATA statistical software (version 11.1; StataCorp, TX, USA) and R software (version 3.2.1; The R Foundation for Statistical Computing). Two-sided *P*≤0.05 was considered significant.

## SUPPLEMENTARY TABLES


