# Supplementary material for: Prognostic value of whole-body SUVmax of nodal and extra-nodal lesions detected by 18F-FDG PET/CT in extra-nodal NK/T-cell lymphoma
Source: Oncotarget. 2016 Dec 10;8(1):1737–43. doi: 10.18632/oncotarget.13873 (PMC5352093; doi:10.18632/oncotarget.13873)
Supplement: Supplementary file 1 [file oncotarget-08-1737-s001.pdf]

Prognostic value of whole-body SUVmax of nodal and extra-nodal lesions detected by ^{18}F-FDG PET/CT in extra-nodal NK/T-cell lymphoma

SUPPLEMENTARY TABLES

Supplementary Table S1: Pairwise comparison of ROC curves in the four models

Pairs	DBA	SE	95% CI	Z-statistics	P-value
SUVmax~ WB1SUVmax	0.16	0.06	0.04~0.27	2.71	<0.001
SUVmax~ WB2SUVmax	0.13	0.05	0.03~0.23	2.56	0.01
SUVmax~WB3SUVmax	0.14	0.05	0.04~0.24	2.71	0.01
WB1SUVmax~WB2SUVmax	0.03	0.02	-0.00~0.06	1.71	0.14
WB1SUVmax~WB3SUVmax	0.02	0.01	-0.01~0.04	1.41	0.16
WB2SUVmax~WB3SUVmax	0.01	0.01	-0.02~0.04	0.46	0.64

DBA, different between areas; SE, standard error.

Supplementary Table S2: L/P-EMD Chemotherapy

	Dose	Route	Day
Methotrexate	3 g/mE+2	IV	1
L/Peg-asparaginase	2500 U/mE+2	IM	2
Dexamethasone	40 mg/d	IV	1, 2, 3, 4
Etoposide	100 mg/mE+2	IV	2, 3, 4

Supplementary Table S3: Nodal and extra-nodal regions for lesion-based analysis in our study

Nodal regions (N=11)	Extra-nodal regions (N=10)
Waldeyer ring	Upper aero-digestive tract
Neck*	Skin/subcutaneous tissues
Infra-clavicular	Central nervous system and spinal canal
Axillary and pectoral	Lung
Mediastinal	Myocardium
Hilar	Bone and bone marrow
Spleen	Bowel
Para-aortic	Renal and adrenal
Mesenteric	Liver
Iliac	Testis
Inguinal and femoral	

*Including cervical, supra-clavicular, occipital, and pre-auricular regions.
